# Loss of Heterozygosity Drives Clonal Diversity of *Phytophthora capsici* in China

**DOI:** 10.1371/journal.pone.0082691

**Published:** 2013-12-12

**Authors:** Jian Hu, Yongzhao Diao, Yuxin Zhou, Dong Lin, Yang Bi, Zhili Pang, Rebecca Trout Fryxell, Xili Liu, Kurt Lamour

**Affiliations:** 1 Department of Plant Pathology, College of Agriculture and Biotechnology, China Agricultural University, Beijing, China; 2 Department of Entomology and Plant Pathology, University of Tennessee, Knoxville, Tennessee, United States of America; Virginia Tech, United States of America

## Abstract

*Phytophthora capsici* causes significant loss to pepper (*Capsicum annum*) in China and our goal was to develop single nucleotide polymorphism (SNP) markers for *P. capsici* and characterize genetic diversity nationwide. Eighteen isolates of *P. capsici* from locations worldwide were re-sequenced and candidate nuclear and mitochondrial SNPs identified. From 2006 to 2012, 276 isolates of *P. capsici* were recovered from 136 locations in 27 provinces and genotyped using 45 nuclear and 2 mitochondrial SNPs. There were two main mitochondrial haplotypes and 95 multi-locus genotypes (MLGs) identified. Genetic diversity was geographically structured with a high level of genotypic diversity in the north and on Hainan Island in the south, suggesting outcrossing contributes to diversity in these areas. The remaining areas of China are dominated by four clonal lineages that share mitochondrial haplotypes, are almost exclusively the A1 or A2 mating type and appear to exhibit extensive diversity based on loss of heterozygosity (LOH). Analysis of SNPs directly from infected peppers confirmed LOH in field populations. One clonal lineage is dominant throughout much of the country. The overall implications for long-lived genetically diverse clonal lineages amidst a widely dispersed sexual population are discussed.

## Introduction

*Phytophthora capsici* Leonian was first described on chili pepper in New Mexico in 1922 [1], and has since been reported on tomato, eggplant, snap and lima beans, and almost all cucurbits [2]. *P. capsici* is a problem worldwide [3-9]. *P. capsici* is an outcrossing organism requiring the interaction of the A1 and A2 mating types to initiate sexual reproduction and formation of thick-walled sexual oospores [10]. *P. capsici* produces massive numbers of deciduous asexual sporangia on the surface of infected plants. Sporangia can germinate directly to cause infection or release 20-40 motile zoospores in free water [6,11]. Under warm and wet conditions, the epidemiology is explosive and entire crops can be lost within a few days [2].

The population structure of *P. capsici* has been characterized at locations worldwide using a variety of genetic markers (e.g. AFLP, SSR and SNP) and appears to vary significantly depending on the location [5,9,12-19]. In the USA and South Africa, populations contain many unique genotypes and sexual reproduction appears to be common [12,14-16,20]. In the USA, spatiotemporal studies suggest clonal lineages do not survive winter or fallow periods and that they are not dispersed widely [14,16,20]. The situation is different in the South American countries of Peru and Argentina where country-specific clonal lineages survive multiple years and are spread widely [17,19]. Similarly, a recent study of *P. capsici* from Gansu province, China indicates three long-lived genetically diverse clonal lineages are widely distributed [18].

Although most *Phytophthora* species are able to reproduce sexually, many are spread widely as clonal lineages including the potato late blight pathogen *P. infestans*, the sudden oak death pathogen *P. ramorum*, and the broad host range pathogen *P. cinnamomi* [21-24]. Mitotic variation within clonal lineages likely plays an important role in their overall evolution and stepwise evolution of new pathotypes has been observed in the oomycete pathogens *P. infestans* [25], *P. sojae* [22] and also other fungal pathogens [26,27]. However, the underlying mechanism(s) for variation within clonal lineages are poorly understood [28]. Mitotic recombination or mitotic gene conversion leading to loss of heterozygosity (LOH) has been reported in several studies [23,28-30], but studies in field populations are limited [31,32]. 

A recent genome project for *P. capsici* produced a high quality reference genome and a genetic linkage map describing inheritance of 20,568 single nucleotide variants [33]. In addition, the genotyping and genetic mapping revealed significant loss of heterozygosity (LOH) had occurred during asexual growth over the course of the project [33]. Loss of heterozygosity refers to a situation that occurs during asexual, mitotic reproduction where areas of the diploid genome carrying heterozygous mutations switch to becoming homozygous. Phytophthora produces massive numbers of asexual spores during a typical infection and this process can potentially lead to isolates carrying diverse genotypes. How the process of LOH occurs in *P. capsici* is unknown. The LOH impacted more than half the single nucleotide variant sites and affected at least 30% of the genome with homozygous tracts ranging in size from 300bp to >1Mbp [33]. In most cases, the LOH was not due to loss of chromosomes and often did not result in obvious injury to the isolates (e.g. slow growth or loss of spore production). The mechanism(s) underlying LOH in *P. capsici* are unknown but LOH was associated with a mating type switch (A2 to A1) and altered virulence and pathogencity in some isolates [33]. The extent or impact of LOH in natural populations is unknown although it is increasingly clear the P. *capsici* genome is highly plastic during in vitro asexual growth and that mating type is an unstable character [18,33].

In this present study, candidate SNP marker assays were developed to investigate the population structure of *P. capsici* in China. Our results suggest significant genotypic variation is produced following sexual and asexual reproduction and that sexual reproduction may be important where winter temperatures are cold. One of our long-term goals is to better inform breeding programs aiming to produce peppers able to withstand attack by *P. capsici*. In the US, where there is an essentially endless stream of unique genotypes produced via sexual recombination, it can be difficult to determine which strains or populations to use to screen promising germplasm. The situation for much of China may be more tractable and testing promising germplasm against a limited number of strains or at a limited number of locations may be useful to identify tolerance or resistance useful on a regional basis. 

## Methods

### Isolate collection and mating type

This work does not include any endangered or protected species. Permission was obtained from all owners of the private land; gardens and farms, to collect plants. During 2006 and 2012, infected tissue samples were collected from multiple provinces in China and isolations were made by plating small sections of infected tissue onto PDA-RPP media (200g potato, 18g dextrose, 12g agar in a total volume of 1liter media amended with 50 ppm of rifampicin, 50 ppm of penicillin, and 50 ppm of pentachloro-nitrobenzene) and incubating at 25 °C for 2 to 4 days [34]. Between one and three isolates were recovered from each plant sample. A single hyphal tip growing from the margin was transferred to new PDA-RPP plates and a unique isolate identifier assigned. For long term storage, 7 mm plugs of actively growing mycelium were transferred into sterile 2 ml microfuge tubes with sterile distilled water and stored at room temperature. 

In addition to individual isolates, infected pepper stem tissue was collected in Guangdong province in 2013 to analyze SNP markers directly from DNA extracted from the infected tissue [35]. Tissue samples were put into 1.5-ml eppendorf tubes within one or two days after collection and stored at -20 °C for at least 1 hour before freeze drying and subsequent genomic DNA extraction (below).

Mating type was determined by co-culturing a 5-mm-diameter plug from a 7-day-old culture with tester isolates of A1 and A2 mating types on V8-RPP agar (as above but substituting 160ml V8 juice for PD) plates (PCAS1 =A1, PCAS2 = A2 were kindly supplied by Mike Coffey from the University of California, Riverside collection). Plates were incubated in the dark for 4-6 days before being checked under a light microscope at 40× magnification for the presence of oospores. Isolates producing oospores with the tester isolate of A1 mating type but not with the A2 tester isolate were determined as the A2 mating type and vice versa, isolates producing oospores with both of the tester isolates were scored as self-fertile (A1/A2) [10]. Isolates selected for genetic diversity analysis were re-tested for mating type prior to genetic analyses. 

### DNA extraction

High quality genomic DNA and crude DNA were used for genotyping. High quality genomic DNA was prepared from mycelium by transferring small wefts of mycelium into 50-ml centrifuge tubes containing 10 mL unfiltered V8-PARP broth (160 mL unfiltered V8 juice, 3 g CaCO3, and 960 ml water amended with 25 ppm pimaricin, 100 ppm ampicillin, 25 ppm rifampicin, and 25 ppm pentachloronitrobenzene).Tubes were incubated horizontally at room temperature for 7 days before mycelium was harvest, lyophilized and powdered as previously described [16]. Genomic DNA was extracted using a standard phenol/chloroform approach [36]. Crude DNA was prepared from mycelium by transferring a small weft of mycelium (approximately 1 mg) to individual wells of a 96-well PCR plate and treating the sample as previously described [35]. For whole genomic DNA, the concentration was estimated by electrophoresis on a 1% agarose gel and using NanoDrop ND-1000 Spectrophotometer (Nano-Drop Technologies, Wilmington, DE, USA). For tissue samples, genomic DNA was extracted as described previously [37].

### Re-sequencing and SNP marker development

Candidate nuclear and mitochondrial SNP markers were identified from 18 isolates of *P. capsici* re-sequenced on an Illumina HiSeq2000 device and aligned to the P. *capsici* reference genome using CLC genome workbench 6.0 (CLC bio, Inc.). Genomic DNA was processed and sequenced at the Children’s Mercy Hospital in Kansas City according to the manufacturer’s instructions (data not shown). Candidate SNPs were selected from the 20568 sites known to have simple Mendelian inheritance and included only those loci which were genic and predicted to be silent [33]. Mitochondrial SNPs were selected based on alignment to a mitochondrial reference genome kindly provided by Dr. Frank Martin. Successful SNP candidates had at least 50 bp free of additional polymorphism up and down-stream of the target site and high resolution DNA melting analysis primers were designed with the LightScanner primer design software 1.0 (Idaho Technology Inc.) to amplify a 45-55 bp amplicon containing the SNP site. 

### High resolution DNA melting analysis (HR-DMA)

HR-DMA genotyping was conducted in 5uL reactions in 384-well plates as previously described [17]. The 5-μL PCR reactions contained 3 μL of genomic DNA (≈3ng/ μL), 0.5 μL 10x buffer, 0.2 μL 5mM dNTPs, 0.05 μL 50 mM MgCl2, 0.025 μL 100μM forward and reverse primers, 0.1 Units Taq Polymerase and 0.5μL 10x LCGreen plus dye (Idaho Technologies, Salt Lake City, UT). The PCR amplification protocol included an initial incubation at 95°C for 2 min, followed by 35 cycles of 95°C for 30 s and 64°C for 30 s and then for duplex formation at 95°C for 30s and a final step at 25°C for 30s. High resolution melting was performed using a 384-well-plate LightScanner device according to the manufacturer’s instructions (Idaho Technology, Salt Lake City, UT). Melt curves were manually assessed to determine homozygous and heterozygous genotypes using the LightScanner 2.0 software. To distinguish homozygous wild and homozygous alternate allele genotypes, an amplicon with a known homozygous genotype was added to each reaction following the initial melt (= doping oligo). The doping oligo was produced in a separate PCR reaction as follows. A synthetic oligo matching one of the possible homozygous sequences was used to PCR amplify larger quantities of the doping oligo. The PCR reaction had a total volume of 30 µL containing 1 μL of 10 μM doping oligo template, 3 μL 10x buffer,1.2 μL 5 mM dNTPs, 1 μL 10 μM forward and reverse primers and 0.3 Units Taq Polymerase. PCR cycling was as above. For the doping reaction, 2 μL of the PCR product was added to the 5 μL genotyping reaction, heated at 95°C for 30 seconds and cooled down to 25°C for 30 seconds for three cycles and melt curves assessed using the LightScanner. Homozygous melt curves changing to heterozygosity following doping are scored as the alternate homozygous genotype. 

### Data analysis

Isolates with missing genotype data were excluded from analyses. Isolates with identical genotypes are designated as MLGX-Y where X identifies a unique multi-locus genotype (MLG) and Y is the number of isolates with identical multi-locus genotypes. Tissue samples with identical multi-locus genotypes are named as TGX_Y. Allele frequencies were calculated based on one representative of each unique genotype. Genetic structure was assessed using Principle Coordinates Analysis (PCA) calculated in GenALEX [38] and Bayesian clustering analysis using STRUCTURE v2.3 [39]. STRUCTURE was run seven times at each K values from 1 to 12 assuming no prior population information, correlated allele frequencies and admixture, 500,000 burn-in cycles and 500,000 Markov Chain Monte Carlo runs (MCMC). The value of K that best fit our data was selected using the ΔK statistic. To estimate genetic differentiation Arlequin 3.5.1.2 was used to calculate population and locus specific pairwise FST for populations within each STRUCTURE hypothesized population [40]. The overall similarity of the isolates (including the year of isolation, number of identical clone-mates, mating type and mitochondrial haplotype) was visualized by building a relationship matrix and heat map using the Population Measures analysis module of JMP Genomics 6.0 (SAS Institute Inc.) with marker ancestry set to “identity by state”.

## Results

### Isolates and mating type

During 2006 and 2012, 1028 isolates of *P. capsici* were collected from 200 locations across 34 provinces of China (Table S1). Most isolates were recovered from pepper except for 6 from pumpkin, 1 from zucchini and 2 from tomato. In total, there were 402 A1 and 619 A2 mating types. In addition, 7 isolates from 2 locations in Fujian province and one location in Shanxi province were self-fertile (A1/A2) (Table S1). In total, 18 of the 27 provinces had both mating types (N = 661), 8 provinces had only the A2 mating type (N = 297) and one province (Hebei) had only the A1 mating type (N= 70). Seven of the 8 provinces with only the A2 mating type (N=277) are located in the southern region of China (Figure S1). 

In 2012, mating type was re-tested for the 276 isolates selected for genetic analysis (Table 1). In total, 7 isolates had a mating type change with 6 changed from the A2 to the A1 and 1 changed from self-fertile to the A1 mating type (Table S2). 

**Table 1:** Summary genetic information for 276 isolates of *Phytophthora capsici* recovered from 2006 to 2012 in China.

Province	Year	Isolates	UG^a^	Mt1^b^	Mt2	Mt3	CL1^c^	CL2	CL3	CL4	NC
Anhui	2006	10	7	6	4	-	3	6	1	-	-
Beijing	2007	7	3	7	-	-	-	3	-	-	4
Fujian	2007, 2009, 2010	17	10	6	11	-	1	-	10	6	-
Gansu	2007, 2009-2011	57	29	36	21	-	20	25	-	11	1
Guangdong	2010-2012	29	12	21	8	-	-	-	8	20	1
Guangxi	2010	10	2	10	-	-	-	-	-	9	1
Guizhou	2010	11	3	11	-	-	-	-	-	9	2
Hainan	2010, 2011	8	6	7	1	-	-	-	1	-	7
Hebei	2007	7	3	6	-	1	-	6	-	-	1
Heilongjiang	2010	7	6	7	-	-	-	-	-	-	7
Henan	2010	7	3	4	3	-	3	4	-	-	-
Hubei	2009, 2010	7	4	5	2	-	-	-	2	5	-
Hunan	2010	9	4	9	-	-	-	-	-	7	2
Inner Mongolia	2007, 2010	6	2	6	-	-	-	4	-	2	-
Jiangsu	2010	6	5	6	-	-	-	3	-	1	2
Jiangxi	2010	3	3	2	1	-	-	-	-	1	1
Jilin	2010	3	3	3	-	-	-	-	-	-	3
Liaoning	2011	8	5	8	-	-	-	1	-	-	7
Qinhai	2011	3	2	3	-	-	-	2	-	1	-
Shandong	2009, 2010	7	5	4	3	-	2	3	-	-	2
Shaanxi	2007	7	4	7	-	-	-	-	-	6	1
Sichuan	2010	10	2	10	-	-	-	-	-	9	1
Tianjin	2009	4	2	-	4	-	4	-	-	-	-
Tibet	2011	5	4	3	2	-	-	2	-	1	2
Xinjiang	2009, 2010	5	2	3	2	-	-	-	-	3	2
Yunnan	2009, 2010	21	4	21	-	-	-	-	-	20	1
Zhejiang	2010	2	1	2	-	-	-	-	-	2	-
	Total	276	-	213	62	1	33	59	22	113	34

^a^ UG= unique genotypes identified based on 39 SNP markers.

^b^ Mt1-3 are three different mitochondrial genotypes.

^c^ CL=clonal lineage, NC=non-clonal.

### SNP markers

Summary data for the 18 re-sequenced isolates of *P. capsici* is presented in Table S3 and the scored heterozygosity for the 20,568 previously described variant sites is presented in Table S4 and includes linkage group, scaffold, variant position, gene model (if genic) and predicted impact of the variant. SNP sites useful for HR-DMA genotyping are free of adjacent mutation and a total of 1872 loci had 50 bp free of polymorphism up and down-stream of the variant site. Of these, 936 loci are predicted to fall within genes and 688 loci are predicted to be silent. In order to increase our chances of developing assays for markers that are polymorphic in China and also possibly useful for populations elsewhere in the world, we focused on the loci that were heterozygous in > 22% of the 18 re-sequenced isolates (N = 437) and selected 48 nuclear loci to design HR-DMA assays and assess genetic variation (Table S5).

**Figure 1:**
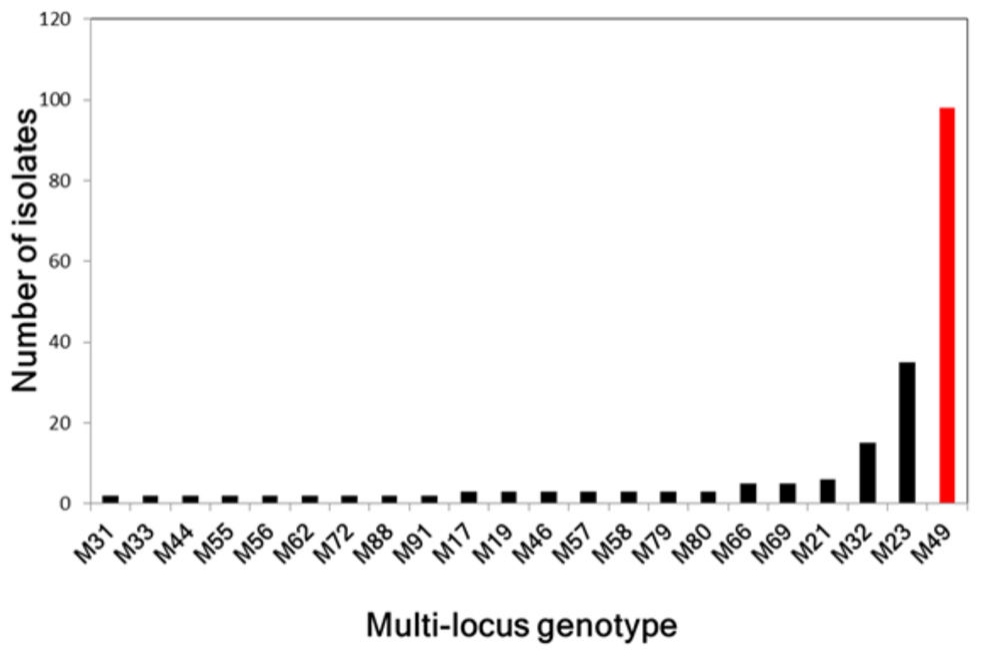
Number of *Phytophthora capsici* isolates within each of the 22 multi-locus genotypes identified in China.

**Figure 2:**
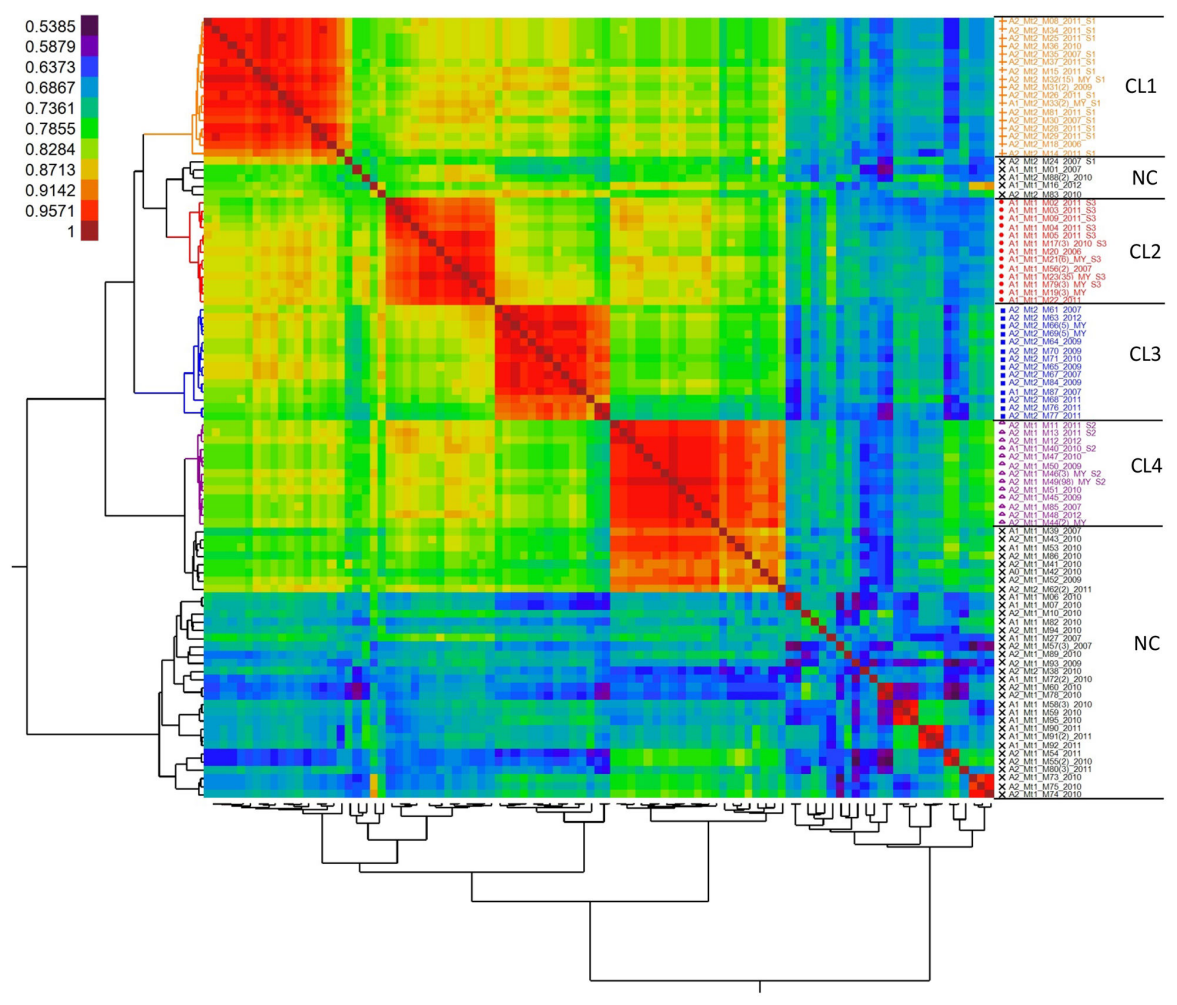
Heat map and dendrogram illustrating genetic diversity of 95 unique genotypes of *Phytophthora* *capsici* recovered from China based on 39 SNP markers. CL=clonal lineage, NC=non-clonal, MY=multiple years. Isolates are denoted with mating type, mitochondrial genotype (Mt), multi-locus genotype (M) and year of collection. The number of the isolates with identical MLGs is listed in brackets if there was more than one. Isolates with an S1, S2 or S3 following the year designation indicate clonal lineages revealed by SSR markers in a previous study of *P*. *capsici* in Gansu province. Genetic similarity is scaled from the least (purple) to the most (red) similar.

Of the 48 markers selected for assay development, 45 were successfully genotyped in all 276 isolates of *P. capsici* from China. Of these, 6 were fixed for homozygosity and 39 were polymorphic (Table 2). The 39 polymorphic loci are found on 35 scaffolds and 15 of the 18 linkage groups of *P. capsici* (Table 2). The 39 polymorphic loci revealed a total of 95 multi-locus genotypes (MLGs) in the 276 isolates (Figure 1). Of these, 73 MLGs were present as singletons and the remaining 22 MLGs had between 2 and 98 isolates with identical genotypes (Table S6 and Figure 2). For all unique genotypes combined, the minor allele frequency varied from 5% to 50% with an average of 34%. 

**Table 2:** Summary data for 39 polymorphic SNP markers used to characterize *P. capsici* in China.

SNP Markera	Linkage Groupb	Allele	Forward primer	Reverse Primer	Protein ID	%c
43_313485	1.0186	C/G	GGCTCCTTTTGGCTCTG	TTAGCACGCGGCTGTTC	535917	44
23_194537	1.0391	C/T	AGGGTTTGGGTGAAGGT	TAATGACAGCCACAAACTCCTC	112886	39
7_1135861	1.163	C/T	CGCGTTCCGCAAGAATG	GCCAGGAACTGCAGGAT	504393	33
41_363483	1.2513	A/G	CTAGGCAACATGGCTCTG	GCTGCACCTTCTTCTCCTG	551335	33
17_164249	1.2864	C/T	ATCGCAAGACAAGCGAG	GTGTTGATTCATATCCAAAACGG	7097	39
13_177577	2.0006	C/T	TACGTCCCCGAGTCTAGAAT	AGCGGATGAAAATGTCCTT	533352	33
11_419981	2.0655	C/T	CGATTAGAACAGCAATCGAGC	CCATCCGAATCGCCAAC	15088	44
30_517298	3.0586	C/T	TTCTAGTCTTCACACGACGAT	CCTCACTGGACAAGGGT	568901	44
37_296971	3.0993	A/G	CTACGGAAGGCCACGCATA	TAGTTGTCCCAGCTCGC	508699	50
22_58618	3.1354	A/G	GACTGCAGAACTTGGCTT	TTGCTGGTGAAGATTCATGTTG	534268	33
10_399503	5.0042	A/G	ATACCTTGGGCCAGGAT	GCACGTTACCACGATGAC	6173	50
24_755911	5.0915	A/G	CCCATGATGTGAACACGATT	CGTAGTGAGAGCCCTAGTTT	507107	50
21_850808	5.1178	A/G	CGACGATCAACGAGTCGCTA	AAGTCCGAGCTGTCCAA	546571	44
16_146633	7.0176	C/T	CCGGGAAGGTCGAAAAG	CCTCCCGGGTTACTGTAG	6989	28
26_474538	8.0258	C/T	GTCGGTCAGCGAACCAT	GGATTGAATACGACACTGAGTGA	8212	39
8_415116	8.0433	A/G	ACTTGTCTACGAAGCTACCGA	GAGCTACCACTGTTGCG	504490	39
22_826381	8.106	A/G	TAAATGGTGTCCGCGCC	GCTCAACCTGCAGCGAA	566837	33
5_78015	9.0031	A/G	AGTTCCACATTGTGTTCACTC	TCCAAGTACGCGCACGA	13811	22
5_914078	9.0343	C/T	TGCTGCAGAACTGTTGGATA	GGTCAAATTGTCGTCCCAT	101441	39
34_147979	10.008	A/C	CTACCGCGTGCATCATT	GGGAGCCTAAAAGCGAAGA	117709	33
40_333183	10.043	A/C	ATCGGAAGCTGTTCGGG	GCTGGTGTCGTTTACCAAG	9660	39
4_507540	10.085	C/T	CTCGAGTCCAAGAGCCT	CACTGCAGATGGCTACG	540026	44
27_18936	10.108	A/G	CCGGCGGACACGTAAAGA	CGTGTTTCTCGTGTCTCATCCA	8263	50
27_139521	10.117	A/G	GAAGCCCTGCAGAAGGAG	TCAATGTAGATGACGTCACCC	115063	50
2_764299	10.157	A/T	CGTAGATAGCAAGGTCTGCCA	CCTTACGCTGCAGGCTC	502951	50
1_1552376	11.012	C/G	GCGCGCGTACCATCCAA	CAGACGCTTCTTGGAGAAAAC	97205	39
32_87587	11.04	A/C	AAGAGCGCGTGGTGTGA	TGTCTTGTGCTGCTGGG	34370	44
29_571713	11.118	C/T	TGCAGCATCTTCACCGT	CATGCGGCATCAGTTGT	64492	67
31_413194	11.134	C/T	TCCAAGTGCTCGGGCTC	GCTTTTGTTTCGGAAGAAAAACCA	64992	56
14_963750	11.147	G/T	CGGCTTGATCCAAGCAAAGA	AGTTCCCACCTCAGGCTAA	108059	33
38_502017	12.031	A/G	TGTGTGCAGTTTCAGTCTGAATA	ACACTCGGCTAGAGAGAAG	508832	50
36_160455	13.005	A/T	GGATTTATTCCACGCCACA	CCTGCAGTATCAAATGCTCT	118503	33
2_679567	13.047	C/T	CCAGTGGAGGACATGGAGAA	CGTCTCGCCTATGAGGT	558677	33
7_136856	13.068	C/T	GGGATGAGTTTGGCGATAC	ACTTTAATTTCCCCAAAGAGGAG	103178	33
42_295125	14.09	A/G	GGGCAATACTTTGGCGG	TGCTCTCGTTCAATCCCAC	18967	44
15_528446	15.022	A/G	CAATACAGAAGCTGCTGAGCA	CAGTACCGCGAGCACCA	564597	39
20_277620	16.01	C/T	GGATTTCTGTACGAACAAGAGC	CCTACCGCCTGCAACATA	506504	50
33_105868	16.063	A/G	GCTCACTGAGTACATTCTCG	CGATCTCCGGTGGGTAG	9004	39
12_58279	18.002	A/C	CTACAAGCAGCGTTAACAC	CTCTCCAGGAAGACGCA	505218	33

^a^ SNP marker are named by their location in the reference genome of *P. capsici* with the first number being the scaffold and the second the polymorphic site.

^b^ The number before the decimal point is the linkage group and the number after the decimal point is used to order each of the 20,568 candidate SNP markers considered in this study.

^c^ The percentage of heterozygous SNP loci in the 18 re-sequenced isolates.

**Figure 3:**
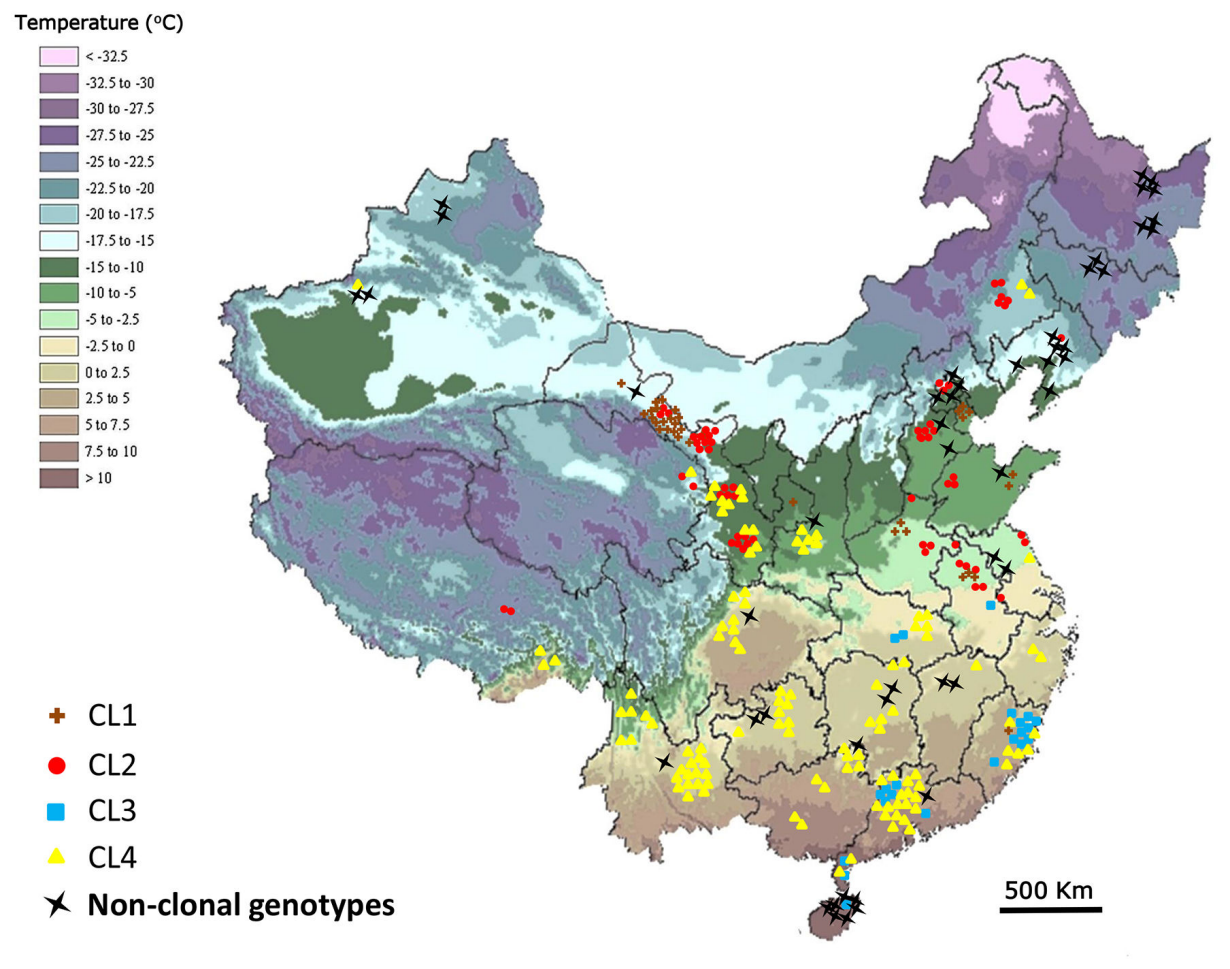
Geographic distribution of clonal lineages (CLs) and non-clonal genotypes in China. The average temperature in January is denoted by the color legend on the top left.

**Figure 4:**
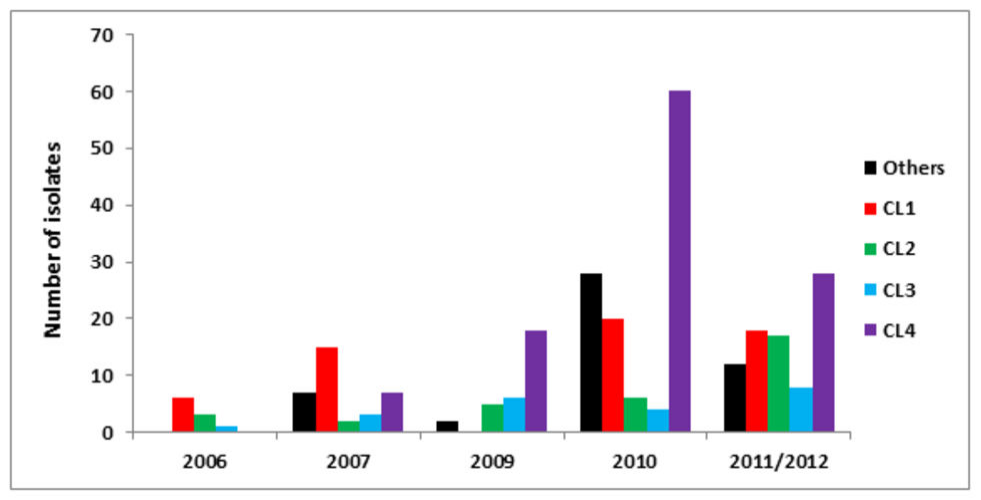
Temporal distribution of clonal lineages (CLs) and non-clonal genotypes from 2006 to 2012 in China.

Isolates with identical, or very similar, multi-locus genotypes comprise clonal lineages and it was obvious that clonally produced strains are wide-spread and long-lived, being found from the far north to the far south and persisting across all 7 years included in the study (Figures 3 and 4). Three mitochondrial haplotypes (Mt1, Mt2 and Mt3) were identified based on the three isolates of *P. capsici* re-sequenced from China. The two SNP assays developed to differentiate the three haplotypes revealed 213 Mt1, 62 Mt2 and 1 Mt3 haplotypes (Table 1). There was no correlation between Mt haplotype and geographical origin. 

### Clonal lineages

A heat map and dendogram illustrating the genetic similarity of the 95 unique multi-locus genotypes indicates isolates cluster into seven distinct groups (Figure 2). Four of the groups contain isolates with high levels of genetic similarity (>85% and often >95%), the same mating type and identical mitochondrial haplotypes. These four genetically similar groups are considered clonal lineages and are referred to as CL1, CL2, CL3 and CL4 (Figure 2). Based on previous findings of extensive LOH in asexually growing isolates of *P. capsici*, we suspect variation within the clonal lineages was most likely due to LOH. CL1 has 17 MLGs (N = 33 isolates) with mitochondrial haplotype Mt2 and all but one are the A2 mating type. The sole A1 mating type had a mating type switch at some point between being tested in China and being re-tested in the US (Table S2). Isolates in CL1 were mainly distributed in the central and west region of China (Figure 3). CL2 has 59 isolates with 13 MLGs, mitochondrial haplotype Mt1 and all isolates are the A1 mating type including two changed from the A2 mating type. CL2 is also widely distributed in the central and west regions of China (Figure 3). CL3 had 22 isolates with 14 MLGs, the Mt2 haplotype and all but one isolate were the A2 mating type with the one exception having a mating type switch from A1/A2 (homothallic) to the A1 mating type (Table S2). CL3 was mainly distributed in the southern region of China (Figure 3). And finally, CL4 was the most common with 13 genetically similar MLGs and 98 isolates having an identical genotype. Isolates in CL4 were mainly distributed in the southern region of China with a few in the west and north (Figure 3). 

The remaining 49 multi-locus genotypes did not group or cluster with any of the above clonal populations and are typical for outcrossing populations of *P. capsici* with both the A1 and A2 mating types present in this group and both mitochondrial haplotypes, although Mt1 is the most common (N = 42). The non-clonal isolates were more commonly found on Hainan Island or in the north region of China (Figure 3). A χ^2^ test for departure from Hardy-Weinberg equilibrium (HWE) for the non-clonal isolates indicates only one SNP marker (7_136856) deviates significantly from expectations based on HWE (P<0.05) (Data not shown).

An assessment of population structure using only the 95 unique multi-locus genotypes using PCA and STRUCTURE indicates five subpopulations corresponding to the 4 clonal lineages (CL1-4) described above and the widely dispersed non-clonal isolates (Figure S2). The five populations were genetically distinct based on pair-wise FST with a values ranging from a low of 0.10 for a comparison of CL1 and CL2 to a high of 0.19 for CL3 and CL4 (*P* < 0.05). An AMOVA test indicates variation is primarily within populations (86.95%) compared to among populations (13.05%). 

### Clonal lineages in infected tissue samples and host specificity

A total of 41 tissue samples from 11 fields were successfully genotyped using 30 markers. This revealed nine genotypes (TG1 to TG9) with four as singletons and five MLG’s having from 2 to 16 members. Genetic similarity analysis indicates all 9 MLG’s are all very similar (>95%) to isolate M49, the most common genotype in the CL4 clonal lineage (Table S7).

Of the 23 isolates of *P. capsici* collected from a single field in Guangdong province in 2012, six were isolated from pumpkin, one from zucchini, two from tomato, and 14 from different varieties of pepper. A total of eight MLGs were identified with two clustering in CL3, five clustering in CL4 and one non-clonal isolate. Isolates in CL3 were recovered from pumpkin and sweet pepper, and isolates in CL4 were recovered from pumpkin, zucchini, tomato and multiple varieties of pepper.

## Discussion

Here we present novel, genetically neutral, SNP markers for *P. capsici* and provide an initial assessment of the population structure of *P. capsici* across China. Worldwide, populations of *P. capsici* differ dramatically for overall diversity with some populations comprised primarily of long-lived clonal lineages whereas other populations appear to undergo annual outcrossing and maintain extensive diversity through sexual recombination. Thus far, it appears either asexual or sexual reproduction drives the overall diversity and population structure within individual countries. For example, in the United States and South Africa, populations are genetically diverse and thick-walled sexual oospores play an important part in the overall epidemiology [12,14-16]. In the South American countries of Peru and Argentina, the situation is markedly different and long-lived clonal lineages persist for years and there is little evidence to suggest sexual recombination is active [19]. A recent study of 279 *P. capsici* isolates recovered from pepper in Gansu province using SSR markers revealed three long-lived clonal lineages dominate the population structure and one of our goals was to determine if the epidemiology of *P. capsici* across the whole of China may be similar to S. America [18]. Because loss of heterozygosity (LOH) can occur once an isolate is brought into axenic culture [33], infected tissue samples were also genotyped to determine if LOH is primarily an artifact due to growing isolates in laboratory culture on agar plates or if LOH occurs during asexual clonal reproduction *in vivo*, in the field setting.

This study includes isolates from 34 provinces of China and includes 28 of the 279 isolates analyzed previously from Gansu province [18]. Overall, the genetic diversity is highly structured with clonal reproduction and clonal lineages causing disease across the entire country. In addition, we found a smaller proportion of genotypically diverse isolates that are not obviously derived from clonal progenitors and are dispersed widely; most commonly in the far north where winter weather conditions are harsh and in the south on Hainan Island where conditions are mild. The overall pattern of genotypic diversity in these areas suggests sexual recombination and outcrossing may be important. In the north, the winter conditions are similar to many vegetable production areas of the mid to northern US and it is possible there is a similar selection pressure for the thick-walled sexually produced oospores for survival [20]. On Hainan Island, the weather is mild year-round and the high proportion of diverse isolates may be due to movement of infected plants or plant parts (e.g. seeds) from the north to the south as part of winter breeding activities [1,41,42]; although additional work is needed to fully understand the situation on Hainan.

Across the mainland of China the population of *P. capsici* resemble S. America and four long-lived clonal lineages are responsible for disease [17,19]. The SNP analysis of the 28 isolates previously reported from Gansu province corroborates the recent findings based on SSR markers – identifying the same three clonal lineages [18]. Furthermore, these clonal lineages are dispersed over wide areas of the country. One lineage is clearly dominant as it is found in every region and comprises >40% of our total sample set. Unfortunately, it is not possible to determine when these clonal lineages became active or where they may have originated. It’s possible the sexual populations in the north (or possibly the south) act as “Phytophthora nurseries” to produce novel genotypes and virulent strains becoming widespread over time [12,43]. How asexual clones of *P. capsici* are spread widely or survive fallow periods is unknown and continued spatiotemporal studies of *P. capsici* in China will be useful to fully elucidate the etiology and fate of these lineages [44]. We found no evidence for host specificity of specific clonal types on pumpkin, zucchini, tomato or multiple varieties of pepper. 

*P. capsici* has an abundance of polymorphic sites with a single nucleotide variant site every 45 bp in the 18 re-sequenced isolates and heterozygous sites, on average, every 200 bp within individual strains [33]. The high density of markers presents opportunities and challenges [45]. One challenge is developing reliable SNP assays useful across multiple populations. In general, SNP-typing assays require contiguous fixed nucleotide sites somewhere near the locus under interrogation. This is true for restriction-enzyme mediated genotype approaches where mutations may obliterate RE sites (e.g. genotype by sequencing (GBS)) [46], assays relying on specific probes (e.g. TaqMan) [47], and strategies requiring PCR amplification like the HR-DMA employed here [16,48]. HR-DMA requires priming sites free of additional mutation flanking the SNP loci and although our data presents only a small sample of the overall diversity for this pathogen, the 18 worldwide isolates allowed us to reduce the 20,568 Mendelian markers to 1872 with potentially polymorphism-free buffers. A focus on sites that are genic and silent (N = 688) and heterozygous in at least a fifth of the isolates (N = 437) made marker selection somewhat easier and there are many additional markers for follow-up or more detailed analyses. 

Overall, the SNP markers revealed a relatively high level of diversity within the clonal lineages. This was not unexpected as recent studies indicate *P. capsici* is capable of dramatic changes during asexual growth in the form of LOH [33]. LOH describes a phenomenon where variable length tracts of genomic DNA spontaneously change to one of the two available haplotypes contained within an individual (diploid) strain [29,49]. In the case of *P. capsici*, the tracts ranged in length from 300 bp to > 1 Mbp [33]. In addition, LOH was associated with loss of pathogenicity and a mating type switch from the A2 to the A1 mating type [33]. Not surprisingly, a similar mating type switch was found for Chinese isolates where the mating type assessed when they were first isolated in China was A2 (or A2/A1) and then switched spontaneously to A1 at some point prior to being re-assessed in the US.

In order to test if the genotypic diversity measured within the clonal lineages reflected diversity extant in the field populations or was possibly due to LOH occurring *in vitro* following isolation onto agar media, SNP genotypes were directly assessed using DNA extracted from infected stem/root tissue for samples collected in 2012. Although the sample set was smaller, it was clear that clonally-related isolates display extensive LOH-driven genotypic diversity in the field and we suspect much of the diversity present in the agar-maintained isolates existed in the field. The frequency and functional impact of LOH within natural populations of *P. capsici* is unknown and will require additional studies aimed at measuring the dynamics of diversity within a clonal framework. 

The implications for an asexual mating type switch from the A2 to the A1 mating type are potentially profound. This allows the formation of thick-walled spores which are able to persist dormant for years. Interestingly, studies looking at the inbreeding potential of *P. capsici* revealed close sibling crosses resulted in apomictic oospore progeny that germinate to produce only one of the parental strains [50]. It’s possible a mating type switch leads to a situation where clone-mates can cross to produce thick-walled apomictic oospores which can survive harsh conditions and allow clonal lineages to persist extended periods.

## Supporting Information

Figure S1 **Mating type distribution of 1028 isolates of *Phytophthora capsici* from 2006 to 2012 in China.** Circles indicate A1 mating type and stars indicate the A2 mating type. The number of isolates with each mating type is listed above the symbol with A1 in red and A2 in blue.
(TIF)

Figure S2 **Principle coordinate analysis (**A**) and STRUCTURE (**B**) analyses of the unique multi-locus genotypes (MLGs) of *Phytophthora capsici* recovered from China between 2006 and 2012.** Members of the four clonal lineages and the remaining non-clonal isolates fall into 5 distinct populations. CL = clonal lineage, NC = non-clonal isolates.
(TIF)

Table S1 **Summary of 1028 isolates of *Phytophthora capsici* collected from 2006 to 2012 in China.**
(DOCX)

Table S2 **Summary genotype data for 276 isolates of *Phytophthora capsici* from China.**
(XLSX)

Table S3 **Summary data for 18 isolates of *Phytophthora capsici* used for whole genome re-sequencing and SNP assessment.**
(DOCX)

Table S4 **Summary data for 20568 SNP sites in 18 strains of *Phytophthora capsici*.**
(XLSX)

Table S5 **Summary data for 48 SNP loci.**
(XLSX)

Table S6 **Summary data for 95 genotypes.**
(XLSX)

Table S7 **Summary data for tissue samples.**
(XLSX)
