# Supplementary figures and images for: Loss of Heterozygosity Drives Clonal Diversity of Phytophthora capsici in China
Source: PLoS One. 2013 Dec 12;8(12):e82691. doi: 10.1371/journal.pone.0082691 (PMC3861455; doi:10.1371/journal.pone.0082691)

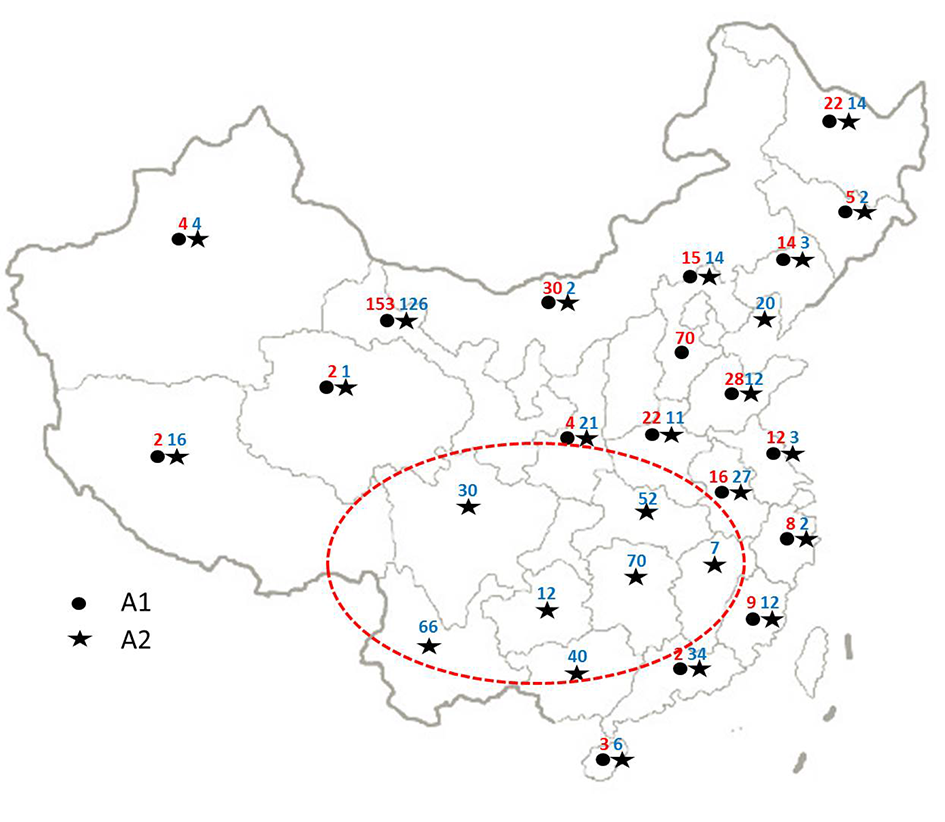

Supplement: Figure S1 — Mating type distribution of 1028 isolates of Phytophthora capsici from 2006 to 2012 in China. Circles indicate A1 mating type and stars indicate the A2 mating type. The number of isolates with each mating type is listed above the symbol with A1 in red and A2 in blue. (TIF) [file pone.0082691.s001.tif]

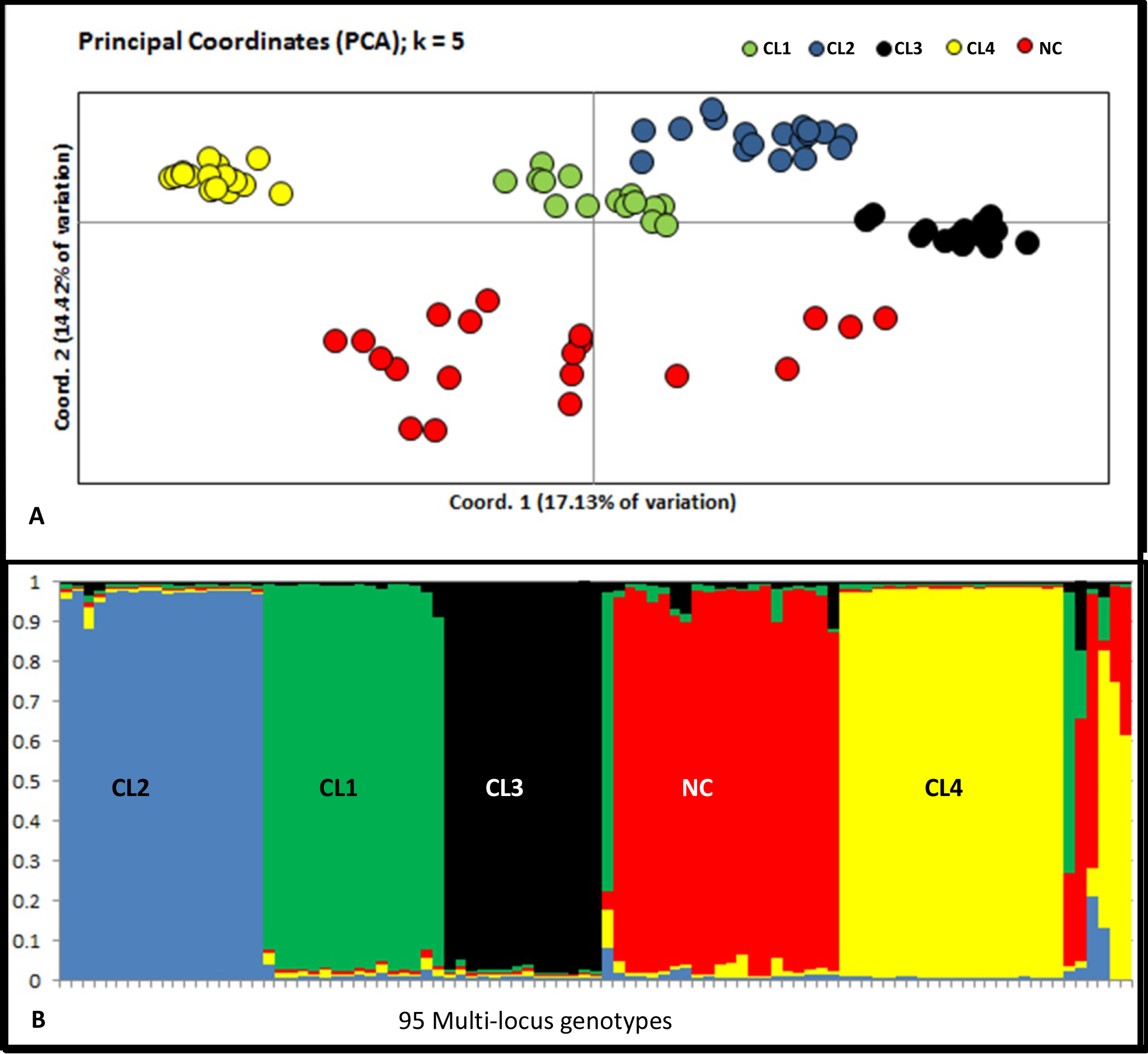

Supplement: Figure S2 — Principle coordinate analysis (A) and STRUCTURE (B) analyses of the unique multi-locus genotypes (MLGs) of Phytophthora capsici recovered from China between 2006 and 2012. Members of the four clonal lineages and the remaining non-clonal isolates fall into 5 distinct populations. CL = clonal lineage, NC = non-clonal isolates. (TIF) [file pone.0082691.s002.tif]
