# Supplementary material for: Loss of Heterozygosity Drives Clonal Diversity of Phytophthora capsici in China
Source: PLoS One. 2013 Dec 12;8(12):e82691. doi: 10.1371/journal.pone.0082691 (PMC3861455; doi:10.1371/journal.pone.0082691)
Supplement: Table S1 — Summary of 1028 isolates of Phytophthora capsici collected from 2006 to 2012 in China. (DOCX) [file pone.0082691.s003.docx]

Table S1. Summary of 1028 isolates of *Phytophthora capsici* collected from 2006 to 2012 in China.

| Province | Number of isolates | Year of isolation | Mating type | | | Source^a^ |
| --- | --- | --- | --- | --- | --- | --- |
|  |  |  | A1 A2 | | A1/A2 |  |
| Anhui | 43 | 2006 | 16 | 27 |  | 1 |
| Beijing | 29 | 2007 | 15 | 14 |  | 2 |
| Fujian | 11 | 2007 | 5 | 2 | 4 | 3 |
|  | 8 | 2009 | 0 | 7 | 1 |  |
|  | 10 | 2010 | 0 | 10 |  |  |
| Gansu | 14 | 2007 | 5 | 9 |  | 4 |
|  | 2 | 2009 | 0 | 2 |  |  |
|  | 118 | 2010 | 67 | 51 |  | 2 |
|  | 137 | 2011 | 68 | 69 |  |  |
| Guangdong | 2 | 2010 | 0 | 2 |  | 2 |
|  | 4 | 2011 | 0 | 4 |  | 5 |
|  | 30 | 2012 | 2 | 28 |  | 4 |
| Guangxi | 40 | 2010 | 0 | 40 |  | 2 |
| Guizhou | 12 | 2010 | 0 | 12 |  | 6 |
| Hainan | 5 | 2010 | 3 | 2 |  | 7 |
|  | 4 | 2011 | 0 | 4 |  | 8 |
| Hebei | 70 | 2007 | 70 | 0 |  | 2 |
| Heilongjiang | 36 | 2010 | 22 | 14 |  | 2 |
| Henan | 33 | 2010 | 22 | 11 |  | 2 |
| Hubei | 24 | 2009 | 0 | 24 |  | 2 |
|  | 28 | 2010 | 0 | 28 |  |  |
| Hunan | 2 | 2009 | 0 | 2 |  | 2 |
|  | 68 | 2010 | 0 | 68 |  |  |
| Inner Mongolia | 30 | 2007 | 30 | 0 |  | 2 |
|  | 2 | 2010 | 0 | 2 |  |  |
| Jiangsu | 15 | 2010 | 12 | 3 |  | 7 |
| Jiangxi | 7 | 2010 | 0 | 7 |  | 2 |
| Jilin | 7 | 2010 | 5 | 2 |  | 9 |
| Liaoning | 17 | 2011 | 14 | 3 |  | 10 |
| Qinhai | 3 | 2010 | 2 | 1 |  |  |
| Shandong | 2 | 2009 | 0 | 2 |  | 11 |
|  | 38 | 2010 | 28 | 10 |  | 2 |
| Shanxi | 25 | 2007 | 2 | 21 | 2 | 2 |
| Sichuan | 30 | 2010 | 0 | 30 |  | 2 |
| Tianjin | 20 | 2009 | 0 | 20 |  | 2 |
| Tibet | 1 | 2009 | 1 | 0 |  | 2 |
|  | 17 | 2011 | 1 | 16 |  | 12 |
| Xinjiang | 3 | 2009 | 0 | 3 |  | 13 |
|  | 5 | 2010 | 4 | 1 |  |  |
| Yunnan | 22 | 2009 | 0 | 22 |  | 14 |
|  | 44 | 2010 | 0 | 44 |  |  |
| Zhejiang | 10 | 2010 | 8 | 2 |  | 2 |
| Total | 1028 |  | 402 | 619 | 7 |  |

^a^ Source of isolates: 1. Rende Qi, Anhui Academy of Agricultural Sciences (Anhui AAS); 2. Xili Liu, China Agricultural University; 3. Furu Chen, FuJian AAS; 4. Heping Lu, Gansu AAS; 5. Birun Lin, Guangdong AAS; 6. Guizhou AAS; 7. Jianxin Wang, Nanjing Agricultural University; 8. Xiaoli Bu, Hainan AAS; 9 Yan Wang, Jilin University; 10, Changyuan Liu, Liaoning AAS; 11, Xiuguo Zhang, Shandong Agricultural University; 12 Wan’an Dai, Xizang AAS; 13, Biao Xu, Xinjiang Agricultural university; 14 Shusheng Zhu, Yunnan Agricultural university.
