# Supplementary material for: Loss of Heterozygosity Drives Clonal Diversity of Phytophthora capsici in China
Source: PLoS One. 2013 Dec 12;8(12):e82691. doi: 10.1371/journal.pone.0082691 (PMC3861455; doi:10.1371/journal.pone.0082691)
Supplement: Table S3 — Summary data for 18 isolates of Phytophthora capsici used for whole genome re-sequencing and SNP assessment. (DOCX) [file pone.0082691.s005.docx]

**Table S3. Summary data for 18 isolates of *Phytophthora capsici* used for whole genome re-sequencing and SNP assessment.**

| **Isolate** | **Year** | **Mating type** | **Country** | **State/Province** |
| --- | --- | --- | --- | --- |
| LT6535 | 2008 | A1 | Argentina | San Carlos |
| LT2135 | 2006 | A2 | Peru | Trujillo |
| LT9397 | 2007 | A1 | China | Hebei |
| LT9288 | 2011 | A2 | China | Gansu |
| LT9400 | 2007 | A1 | China | Hebei |
| LT7395 | 2010 | A1 | France | Eygalieres |
| LT7704 | 2010 | A1 | France | - |
| LT7701 | 2010 | A1 | France | - |
| LT6745 | 2002 | A1 | Mexico | Michoacán |
| LT263 | 2004 | A2 | USA | Tennessee |
| LT51 | 1997 | A1 | USA | Michigan |
| LT62 | 1998 | A2 | USA | Michigan |
| LT72 | 1998 | A1 | USA | Michigan |
| LT6503 | 2009 | A2 | USA | Connecticut |
| LT9417 | 2011 | A1 | USA | South Carolina |
| LT9418 | 2011 | A1 | USA | South Carolina |
| LT9415 | 2011 | A1 | USA | Wisconsin |
| LT6149 | 2008 | A2 | USA | Long Island, NY |
